# Is multi-joint hip and knee osteoarthritis more than the sum of its parts?

**DOI:** 10.1016/j.ocarto.2026.100814

**Published:** 2026-05-14

**Authors:** M.A. van den Berg, E. Panfilov, S.M.A. Bierma-Zeinstra, J.H. Krijthe, R. Agricola, A. Tiulpin

**Affiliations:** a Department of Orthopaedics and Sports Medicine, Erasmus Medical Center, Rotterdam, the Netherlands; b Research Unit of Health Sciences and Technology, University of Oulu, Oulu, Finland; c Department of Diagnostics, Oulu University Hospital, Oulu, Finland; d Department of General Practice, Erasmus Medical Center, Rotterdam, the Netherlands; e Pattern Recognition and Bioinformatics Group, EEMCS, Delft University of Technology, Delft, the Netherlands; f Department of Radiology, Weill Cornell Medicine, New York, USA

**Keywords:** Multi-joint osteoarthritis, Patient stratification, Joint space width narrowing, Holistic approach

## Abstract

**Objective:**

Osteoarthritis (OA) is typically studied in isolated joints, but humans are interconnected systems. This raises the question of how multi-joint OA manifests, and whether it forms a distinct subgroup. This study aimed to investigate whether individuals with OA worsening in both the hip and the knee exhibit unique clinical, structural, or demographic characteristics compared to those with isolated OA worsening or no worsening.

**Design:**

We conducted a retrospective analysis using data from the Osteoarthritis Initiative, including 1958 participants with radiographic assessments of hip and knee joints at baseline and 48-month follow-up. Participants were categorized into four groups based on joint space narrowing: no worsening, hip-only worsening, knee-only worsening, or combined worsening in 48 months. Univariate comparisons and multivariate logistic regression analyses were performed to compare the combined worsening group to the other groups.

**Results:**

Combined worsening occurred in 12.5% of participants. Compared to those with no worsening, the combined worsening group had more severe baseline radiographic knee OA (aOR: 1.38 (1.15–1.64)). Compared to hip-only OA worsening, the combined group had more severe knee OA (aOR: 1.36 (1.11–1.67)). Compared to those with knee-only OA worsening, combined OA worsening was associated with female sex (aOR: 1.92 (1.31–2.76)).

**Conclusions:**

Our findings show differences between individuals with combined or isolated OA worsening, which may reflect accumulation of single-joint risk factors rather than a distinct trajectory. This research provides a foundation for large-scale investigations into multi-joint OA subtypes to improve patient stratification and inform targeted interventions.

## 1 Introduction

Osteoarthritis (OA) commonly affects the hip and knee, often resulting in pain, functional limitations, and reduced quality of life [1,2]. Beyond its personal impact, OA imposes a significant burden on healthcare systems and the economy [3]. While both the hip and knee are frequently studied in OA research due to their high prevalence and clinical impact, most OA studies focus on these joints in isolation, potentially overlooking important patterns of multi-joint disease worsening within individuals [4,5].

To date, the reductionist consideration of OA as a single-joint joint disease dominates despite the known biomechanical and systemic links between the hip and knee joints [[6], [7], [8]]. For instance, altered load distribution or compensatory movement patterns resulting from OA in one joint may add stress to and degeneration of the contralateral or adjacent joints [9,10]. Moreover, systemic contributors such as inflammation, metabolic dysregulation, and psychosocial factors are increasingly recognized as potential drivers of multi-joint OA worsening, underscoring the need for a more integrated approach to OA characterization [11].

Understanding how structural changes, as well as demographic and clinical factors might differ for individuals with combined hip and knee OA worsening is essential not only for improving prognostic models but also for tailoring clinical interventions and trial designs to reflect real-world multi-joint disease patterns. Supporting the relevance of investigating multi-joint OA, Nelson et al. cross-sectionally studied a small cohort of participants recruited for general OA research and reported that one in five individuals had radiographic OA (ROA) in more than one joint, with hip and knee OA co-occurring in one in ten cases [12]. However, it remains unclear whether individuals with combined radiographic worsening can be reliably distinguished from those with isolated radiographic worsening using routinely collected data in a larger cohort. This distinction could have implications for clinical trial design, including the selection of inclusion criteria and interpretation of outcomes.

This exploratory study addresses this gap by comparing the clinical, structural, and demographic profiles of individuals with combined hip and knee OA worsening to those with hip-only, knee-only, or no worsening. We conduct both univariate and multivariate analyses to explore the potential differences comprehensively and to inform future understanding of osteoarthritis as a whole-person disease.

## 2 Method

### 2.1 Study design and population

The retrospective cohort study was conducted and reported in line with the Strengthening the Reporting of Observational Studies in Epidemiology guidelines [13]. Data were obtained from the Osteoarthritis Initiative (OAI, https://data-archive.nimh.nih.gov/oai/), a multi-center, longitudinal cohort study of participants aged 45–79 years at baseline who either had or were at increased risk of developing knee osteoarthritis. Written informed consent was obtained from all participants prior to their enrollment in the OAI.

For the present study, we included participants with complete bilateral posteroanterior knee radiographs and standardized weight-bearing anteroposterior pelvic radiographs acquired at both the baseline and 48-month follow-up visits. Participants were excluded if they had missing ROA grades or a hip or knee replacement at baseline. To enable definition of the worsening groups, participants were included if they had minimum joint space width (mJSW) measurements for both knees available through the original OAI dataset. Additionally, the pelvic radiographs had to be available and of high enough quality for an in-house developed automated hip mJSW measurement protocol. It must be noted that in OAI the mJSW readings were performed for all participants in their progression and non-exposed control sub cohorts, but in the incidence cohorts, only the knees with ROA (KLG≥2) and a subset of those without ROA were measured.

### 2.2 Definition of worsening groups

For knee joints, mJSW in the medial tibiofemoral compartment was measured using a semi-automated software tool that delineated the femoral and tibial contours to find the minimum distance, expressed in millimeters [14,15]. Both the baseline and 48-month radiographs were read in pairs, blinded to chronological order, with manual verification and corrected as needed. The medial tibiofemoral mJSW measurements were available and validated as a standard part of the OAI dataset, where the test-retest reliability for change in mJSW from baseline to 36 months showed an intraclass correlation coefficient of 0.955 and limits of agreement of −0.522 to 0.401 mm. Given the consistent measurement methodology, this was assumed to also apply to 48-month data.

**Fig. 1:**
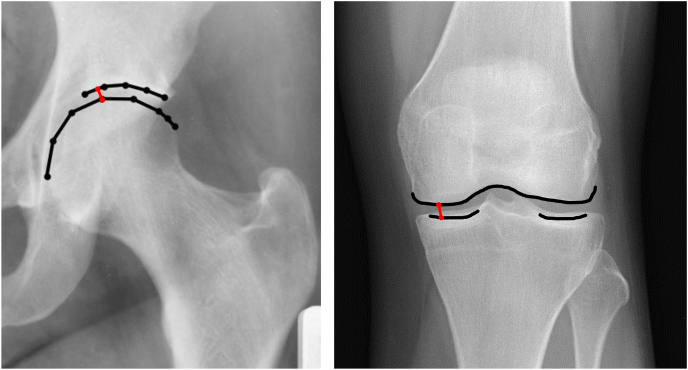
Example of the minimal joint space width measurements in the hip joint (left, including automatically placed landmark points) and the medial compartment of the knee joint (right).

For hip joints, mJSW was defined as the minimal distance (in mm) between the contours of the femoral head and weight-bearing part of the acetabulum. These contours were drawn between automatically placed landmark points with the Bonefinder® software using linear B-spline interpolation (Fig. 1). Baseline and follow-up pelvic radiographs were analyzed blinded to chronological order. The standardized weight-bearing anteroposterior pelvic radiographs were obtained at both timepoints using a V-shaped foot-positioning frame to place the feet in 5° of internal rotation. The landmark protocol has been previously published and has been developed within the World COACH consortium [16,17]. To ensure data quality, a random subset of images was manually reviewed.

At an individual level, radiographic knee OA (RKOA) worsening was defined as a reduction in mJSW of ≥0.7 mm in at least one knee, and radiographic hip OA worsening was defined as a reduction in mJSW of ≥0.5 mm in at least one hip over the 48-month interval. The chosen thresholds were informed by prior evidence and expected ROA grade-dependent trajectories and their associations with future symptoms and/or interventions [[18], [19], [20], [21], [22]]. Additionally, presence of any total knee replacement or any total hip replacement at the 48-month visit was considered OA worsening for the respective joint type. Participants were then categorized into having no worsening, hip-only worsening (any hip), knee-only worsening (any knee), or combined worsening (at least one hip and one knee).

### 2.3 Considered covariates

To compare the characteristics of individuals across the four OA worsening groups, a range of baseline variables were considered. These variables were selected based on their relevance to OA risk, structural joint integrity, systemic involvement, and potential genetic predisposition.

#### 2.3.1 Baseline demographics

Age, birth-assigned sex, and Body Mass Index (BMI) were included as core demographic variables, given their established association with OA incidence and progression [23].

#### 2.3.2 Clinical features

Clinical features included self-reported knee and hip pain, assessed via the OAI symptom questionnaires. Pain was defined as the presence of frequent pain, aching, or stiffness in either the right or left joint on most days of the past month. Additionally, knee injury status was categorized into three groups based on participant responses: (1) no history of knee injury, (2) history of knee injury without surgery, and (3) history of knee surgical intervention regardless of injury. These categories were designed to capture both the occurrence of injury and whether it led to a medical intervention, which may reflect different pathways of joint damage [24,25]. To account for systemic health factors, we included the presence of comorbidities such as asthma, other chronic lung diseases, diabetes, and rheumatoid arthritis [26]. Abdominal circumference was also considered as a proxy for central adiposity, which may reflect underlying metabolic or inflammatory processes relevant to OA pathophysiology [27].

#### 2.3.3 OA history and genetic indicators

Indicators of broader OA involvement and potential genetic predisposition were captured through self-reported presence of hand OA, and family history of hip or knee replacement surgery [[28], [29], [30]].

#### 2.3.4 Structural joint features

Joint-specific OA status was assessed on person level using baseline Kellgren-Lawrence (KL) grades for the knee [31] and by the OAI defined ROA grade classification system for the hip [32]. The hip ROA classification was based on a three-level system: no OA, possible OA, and definite OA, derived from radiographic features including joint space narrowing and osteophyte presence. To describe OA status for the hip and knee joint on a person level, the highest grade observed in either the left or right joint for each knee or hip was selected.

To further characterize joint morphology relevant to biomechanics, we incorporated measures of knee flexion contracture, knee alignment angle, hip alpha angle (AA) and hip lateral center-edge angle (LCEA). These shape-related parameters were considered as potential modifiers of mechanical loading and joint degeneration [[33], [34], [35], [36]]. Additionally, baseline mJSW was considered to adjust for pre-existing differences in JSW and to investigate potential cross-joint effects.

### 2.4 Statistical analysis

All analyses were performed using R (version 4.3.2, R foundation for Statistical Computing, Vienna, Austria) [37]. Continuous variables are presented as mean with standard deviation, and categorical variables are presented as counts and percentages.

To examine differences in covariates between the combined worsening group and each of the other groups, univariate analyses were performed. Continuous variables were compared using the Student's t-test after assessing approximate normality, while categorical variables were compared using the chi-square or Fisher's exact test, as appropriate. A total of 28 covariates were tested across three pairwise comparisons to the combined worsening group as reference (84 tests in total). The significance level was set to 5% to control the Type I error rate. To adjust for multiple testing, Hochberg's step-up procedure was applied to control the family wise-error rate [38].

To assess the independent effects of multiple variables simultaneously, a multinomial logistic regression model was fitted using the combined worsening group as reference. Predictor selection for the multivariate analysis was guided by prior literature on OA risk factors and aimed to represent distinct domains: demographics, structural features, and clinical history. Variables with substantial missing data were excluded. Additionally, predictors that overlapped conceptually with other included variables were deprioritized. The continuous predictors were standardized to zero mean and unit variance based on the overall mean and standard deviation of the entire included study population. Only the OA status in the hip and knee was modeled continuously using the original grade scales (0–2 for the hip, 0–4 for the knee). The primary analyses were conducted as complete-case analyses, including only individuals with complete data for all predictors. To assess the robustness of the findings to missing data, we also performed a sensitivity analysis using multiple imputation. Adjusted odds ratios (aORs) and their 95% confidence intervals were estimated using bootstrapping with 10,000 iterations. To improve the reliability of our effect estimates, we applied Bias-Corrected and Accelerated bootstrapping [39], a method that adjusts for bias and skewness in the sampling distribution. This approach is particularly effective where data may be skewed or has a small size, enabling more precise estimates of uncertainty in aORs [39]. The inverse aORs were reported to interpret them using the combined worsening group as positive class. Finally, the explained variance of the multinomial model was assessed through the Nagelkerke R^2^, and improvements in model fit over an intercept-only model were evaluated using the likelihood ratio test [40].

## 3 Results

### 3.1 Study population

**Fig. 2:**
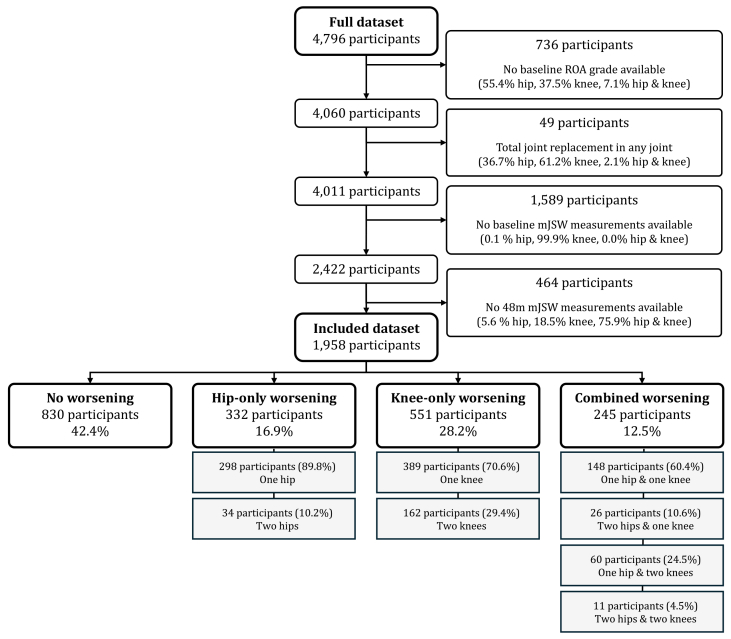
Flow of participants from the full OAI dataset to the final included set of participants, split into the four worsening groups. ROA: Radiographic osteoarthritis, mJSW: minimum joint space width, 48 m: 48 months.

A total of 1958 participants met all inclusion criteria and were classified into four OA worsening groups based on radiographic JSN over 48 months: no worsening (42.4%), hip-only worsening (16.9%), knee-only worsening (28.2%), and combined worsening (12.5%). Fig. 2 provides a detailed overview of the inclusion/exclusion flow and the distribution of joint involvement in OA worsening. While the availability of knee JSW measurements was limited to a subset of participants, we assessed the potential impact of this selection bias by comparing baseline characteristics between included and excluded individuals. These comparisons, detailed in Supplementary Table S1, showed that except for the baseline OA status, differences were generally small, suggesting that the included sample remains broadly representative of the larger cohort. Additionally, a logistic regression model predicting missingness based on mJSW measurement availability (Supplementary Table S2), supports the notion that the baseline RKOA grade was the primary factor influencing participant exclusion. More specific details on the population's baseline ROA status and JSN in the four different joints can be found in Supplementary Table S4–S6.

### 3.2 Univariate analysis between the combined worsening groups & other groups

Using the combined worsening group as a reference, univariate comparisons revealed several notable differences (Table 1). Participants in the combined worsening group had significantly more severe baseline RKOA grades compared to both the no worsening and hip-only worsening groups (p < 0.0001). Additionally, baseline hip mJSW was slightly but significantly larger in the combined group compared to the no worsening and knee-only worsening groups.

**Table 1:** Demographics and baseline characteristics by worsening group. Additionally included are the p-values of pairwise comparisons with the combined worsening group.

Variable	Worsening group			
	No	Hip-only	Knee-only	Combined
Number of participants, N (%)'	830 (42.4)	332 (16.9)	551 (28.2)	245 (12.5)
**Demographics**				
Age [years], mean (SD)	61.10 (9.02)	60.91 (9.18)	61.61 (9.04)	61.47 (8.34)
BMI [kg/m^2^], mean (SD)	28.51 (4.54)	28.29 (4.81)∗	29.34 (4.53)	29.34 (4.40)
Female participants, N (%)''	515 (62.0)	211 (63.6)	272 (49.4)	136 (55.5)
**Clinical factors**				
Hip pain present, N (%)''	438 (53.0)	189 (57.4)	290 (52.8)	134 (54.9)
Knee pain present, N (%)''	677 (81.7)∗	277 (83.7)	485 (88.0)	216 (88.5)
Knee injury status, N (%)''	∗∗	∗		
No injury	438 (53.3)	166 (50.8)	252 (46.1)	102 (42.1)
Injury without surgery	204 (24.8)	92 (28.1)	140 (25.6)	67 (27.7)
Surgical intervention	179 (21.8)	69 (21.1)	155 (28.3)	73 (30.2)
Asthma, N (%)''	57 (7.0)	26 (8.0)	55 (10.2)	13 (5.5)
Lung disease, N (%)''	12 (1.5)	7 (2.1)	10 (1.9)	3 (1.3)
Diabetes, N (%)''	59 (7.2)	22 (6.8)	46 (8.5)	19 (7.9)
RA, N (%)''	12 (1.7)	2 (0.7)	11 (2.4)	6 (2.9)
Abdominal circumference [cm], mean (SD)	102.84 (11.93)	103.13 (13.12)	103.75 (12.42)	104.00 (11.67)
**OA history and genetic indicators**				
Hand OA, N (%)''	129 (16.2)	57 (17.9)	80 (15.1)	35 (14.6)
Family member with hip replacement, N (%)''	73 (8.8)	31 (9.5)	45 (8.2)	27 (11.2)
Family member with knee replacement, N (%)''	115 (14.0)	34 (10.6)	87 (16.1)	36 (15.1)
**Structural joint features**				
Hip OA status in worst hip, N (%)''				
No OA (grade 0)	638 (76.9)	245 (73.8)	419 (76.0)	183 (74.7)
Possible OA (grade 1)	129 (15.5)	48 (14.5)	76 (13.8)	43 (17.6)
Definite OA (grade 2)	63 (7.6)	39 (11.7)	56 (10.2)	19 (7.8)
Knee OA status in worst knee, N (%)''	**∗∗∗∗**	**∗∗∗∗**		
KLG 0	144 (17.3)	50 (15.1)	45 (8.2)	27 (11.0)
KLG 1	122 (14.7)	55 (16.6)	50 (9.1)	18 (7.3)
KLG 2	339 (40.8)	130 (39.2)	201 (36.5)	86 (35.1)
KLG 3	163 (19.6)	74 (22.3)	208 (37.7)	85 (34.7)
KLG 4	62 (7.5)	23 (6.9)	47 (8.5)	29 (11.8)
Left hip				
AA [degrees], mean (SD)	49.24 (12.58)	48.97 (12.52)	50.70 (12.37)	49.11 (12.29)
LCEA [degrees], mean (SD)	36.18 (5.65)∗∗	35.30 (5.99)	35.61 (5.59)∗	34.87 (5.86)
min JSW [mm], mean (SD)	**3.40 (0.56)∗∗∗∗**	3.67 (0.58)	**3.44 (0.58)∗∗∗∗**	3.79 (0.63)
Right hip				
AA [degrees], mean (SD)	47.64 (11.55)	47.33 (11.05)	49.05 (11.79)	47.91 (11.24)
LCEA [degrees], mean (SD)	35.10 (5.55)∗∗	34.18 (5.45)	34.79 (5.61)∗∗	33.97 (5.72)
mJSW [mm], mean (SD)	**3.46 (0.56)∗∗∗∗**	3.76 (0.55)	**3.48 (0.58)∗∗∗∗**	3.86 (0.68)
Left knee				
Flexion contracture/hyperextension (contracture positive) [degrees], mean (SD)	−0.32 (4.22)	−0.12 (4.22)	0.55 (4.12)	0.13 (4.12)
Alignment angle (Valgus negative) [degrees], mean (SD)	−0.78 (3.91)	−1.15 (3.30)∗∗	−0.04 (4.04)	−0.26 (3.89)
mJSW [mm], mean (SD)	4.17 (1.55)	4.27 (1.15)	4.12 (1.55)	4.18 (1.19)
Right knee				
Flexion contracture/hyperextension (contracture positive) [degrees], mean (SD)	0.21 (3.97)	0.44 (4.26)	1.17 (4.15)	0.85 (4.40)
Alignment angle (Valgus negative) [degrees], mean (SD)	−0.62 (3.77)∗	−1.09 (3.44)∗∗∗	−0.01 (4.02)∗	−0.08 (3.85)
mJSW [mm], mean (SD)	4.17 (1.47)	4.28 (1.13)	4.33 (1.50)	4.20 (1.17)

AA: Alpha angle, BMI: Body Mass index, LCEA: Lateral Center Edge Angle, mJSW: minimum joint space width, KLG: Kellgren & Lawrence grade, OA: Osteoarthritis, RA: Rheumatoid arthritis.

P-values are calculated through pairwise *t*-test for the continuous variables and chi-square or Fisher's extract for the categorical variables.

Significance level compared to combined worsening group: ∗ = p < 0.05, ∗∗ = p < 0.01, ∗∗∗ = p < 0.001, ∗∗∗∗ = p < 0.0001.

Underlined and bold entries indicate significant differences with the combined group after adjustment of multiple testing through Hochberg's step-up procedure.

‘ Percentage is relative to full dataset.

’’ Percentage is relative to available data within this worsening subgroup.

Although not statistically significant after adjustment for multiple testing, several variables showed suggestive trends. BMI appeared slightly higher (+1 kg/m^2^) in the combined worsening group compared to the no and hip-only groups. The proportion of female participants was lower in the combined group (55.5%) relative to the no worsening (62.0%) and hip-only worsening (63.6%) groups, while the knee-only group had nearly equal sex distribution. Knee pain was slightly more prevalent in the combined worsening group (88.5%) compared to the no (81.7%) and hip-only (83.7%) worsening groups, but comparable to the knee-only (88.0%) worsening group. Overall, the low prevalence of comorbidities across all worsening groups made comparisons in this subgroup of variables challenging. A higher proportion of the combined worsening participants did have family members with a hip replacement compared to all other groups. Knee injury status also varied, with the highest proportion of participants reporting prior knee injuries in the combined group (57.9%).

### 3.3 Multivariate analysis

Baseline predictors for the multinomial regression multivariate models were selected to represent distinct domains: demographics, structural features, and clinical history. Selection was further guided by data availability and having at least 10 positive cases per predictor. The final model included age, BMI, birth-assigned sex, knee injury status (binarized into injury vs no injury), hip pain, knee pain, family history of hip or knee replacement surgery, knee alignment angle, ROA status, and mJSW as baseline predictors of interest. Correlation coefficients between these variables are provided in Supplementary Fig. S1. In total, complete data of 1807 individuals was available for this analysis. Supplementary Table S3 summarizes the key characteristics for the included as excluded (n = 151) participants. The model showed improved fit over an intercept-only model (likelihood ratio test, p ≪ 0.00001) and the Nagelkerke's R^2^ of 0.158 indicates that approximately 15.8% of the variance in the outcome is explained by the model.

**Fig. 3:**
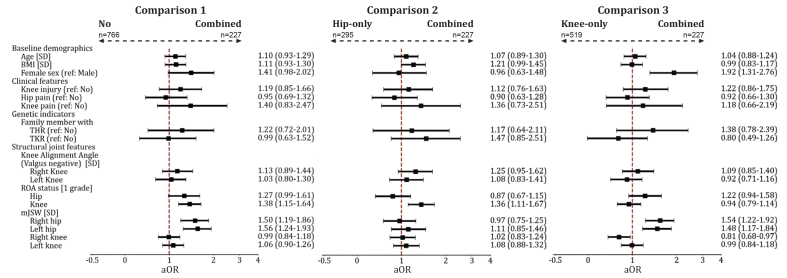
Forest plots displaying adjusted odds ratios (aORs) and bootstrapped 95% confidence intervals estimated with a multinomial logistic regression model for predictors of combined worsening compared to no worsening (Comparison 1), compared to hip-only worsening (Comparison 2), and compared to knee-only worsening (Comparison 3). The x-axis is presented on a logarithmic scale. BMI: Body Mass Index, mJSW: minimum joint space width, ROA: radiographic osteoarthritis, SD: standard deviation, THR: total hip replacement, TKR: total knee replacement.

An overview of aORs and BCa bootstrapped confidence intervals for all predictors across the three comparisons is presented in Fig. 3. First, we compared participants with combined worsening (n = 227) to no worsening (n = 766), representing differences between individuals with no OA worsening to those with worsening in both the hip and knee. We found an association with more severe knee ROA for combined versus no worsening, which was linked to a 38% increase in the odds of combined worsening per grade (aOR = 1.38, 95% CI: 1.15–1.64). Additionally, higher baseline mJSW values in hips were associated with increased odds of combined OA worsening. Secondly, we compared the combined worsening group (n = 227, 43% of included individuals in comparison 2) to participants with hip-only worsening (n = 295), focusing on factors that may distinguish individuals with hip progression who also show signs of knee involvement. In this comparison, the presence of definite ROA in the knee was associated with increased odds of combined worsening. Third, we compared participants with combined OA worsening (n = 227, 30% of included individuals in model 3) to those with knee-only worsening (n = 519), examining what factors may indicate whether individuals with knee worsening also had hip worsening. Female sex was associated with increased odds of combined worsening, indicating that they were almost twice as likely to experience progression in both joints compared to men (aOR = 1.92, 95% CI: 1.31–2.76). Additionally, higher baseline mJSW in the hip joints were associated with increased odds of combined worsening. Across all three comparisons, the findings from the complete-case analysis were supported by sensitivity analyses using multiple imputation for missing predictor data, where differences in aOR were smaller than 0.1 (Supplementary Table S7).

## 4 Discussion

In this study, we have investigated whether individuals with combined hip and knee OA worsening over 48 months exhibit distinct clinical, structural, or demographic profiles compared to those with isolated joint worsening or no worsening. This group represented approximately 12.5% of the OAI cohort. Within this high-risk population for knee OA, 30% of participants exhibited hip worsening within 48 months, highlighting the prevalence and clinical significance of investigating multi-joint involvement. While some differences between the combined group and the no or isolated worsening groups were observed, particularly in baseline ROA severity and structural features, these patterns were not uniformly strong or consistent across all comparisons. Nonetheless, our findings highlight that multi-joint OA progression may be influenced by female sex and baseline joint morphology.

We first examined whether individuals with combined hip and knee OA worsening differ meaningfully from those who showed no radiographic progression in either joint. While descriptive differences in BMI and OA severity were noted, only definite knee ROA showed a statistically significant association with combined worsening (aOR = 1.38, 95% CI: 1.15–1.64) in the multivariate analysis. Baseline mJSW in the hip also emerged as a relevant variable, though its association may partly reflect regression to the mean effects due to its role in defining the outcome. It is important to note that the “no worsening” group likely includes a heterogeneous mix of individuals, some with stable ROA and others without ROA at baseline, which may dilute potential differences.

Subsequently, we have explored within the population with hip OA worsening whether individuals who also experienced knee OA worsening differed from those with hip-only worsening. This analysis focused on identifying factors that are associated with individuals that have hip OA worsening with simultaneous knee OA worsening. The presence of definite ROA in the knee was associated with increased likelihood of combined worsening (aOR = 1.36, 95% CI: 1.11–1.67). Additionally, higher BMI showed a trend towards significance with an aOR of 1.21 (95% CI: 0.99–1.45). While both are well established risk factors for knee OA worsening, it remains unclear whether elevated BMI contributes to the multi-joint worsening through systemic mechanisms, such as low-grade inflammation and metabolic dysregulation, or primarily through mechanical overloading of the knee joint [3]. The lack of a clear link between BMI and hip OA worsening, in this context, raises the possibility that BMI might be acting more as a knee-specific risk factor rather than a driver for multi-joint disease. Future research is needed to disentangle these pathways and determine whether individuals with hip OA worsening might also have knee OA worsening due to shared systemic exposures or joint-specific vulnerabilities [41].

In our final series of experiments, we have investigated whether individuals with knee OA worsening who also experienced hip worsening differed from those with knee-only worsening. Here, female sex and higher baseline hip mJSW were associated with increased odds of combined worsening. The association with female sex is particularly noteworthy, as previous studies have shown that women are more likely to experience symptomatic hip OA than men, even though radiographic hip OA tends to be more prevalent in men [42]. Our findings highlight that, among those with knee OA worsening, women may be more likely to also experience hip OA worsening. On one hand, it might reflect a sex-specific vulnerability to hip osteoarthritis specifically based on differences in anatomy. On the other hand, this could also reflect a sex-specific vulnerability to multi-joint OA worsening, potentially influenced by hormonal dysregulation i.e., post-menopausal effects, which warrants further investigation.

Beyond our multivariate comparisons, one characteristic that stood out in the univariate analysis was the history of knee injury. A higher proportion of individuals in the combined worsening group reported prior knee injuries, supporting previous findings that joint trauma may predispose individuals to more aggressive OA progression, potentially through altered biomechanics or inflammatory responses [[43], [44], [45]]. However, this association did not persist in the multivariate models, suggesting that knee injury status may not independently predict multi-joint OA worsening when other factors are considered. Still, its prominence in the descriptive data highlights the need for further exploration, particularly in studies designed to assess the long-term impact of joint trauma across multiple joints.

Several limitations of this study should be acknowledged. First, the definition of OA worsening was based on JSN derived from two-dimensional radiographic imaging. While JSN is a widely used marker of structural progression, it may be influenced by joint positioning and image resolution. The reproducibility of mJSW measurements can vary [46], and subtle changes over time may be difficult to detect reliably. Future studies should consider incorporating more advanced biomarkers, such as fixed JSW or joint convergence line metrics, which may offer improved sensitivity and anatomical specificity [47]. Second, the OAI dataset included participants at risk of knee OA and the availability of knee JSW data was limited to a subset of healthy controls, potentially introducing selection bias towards more participants with severe knee OA. Although baseline comparisons between included and excluded individuals based on mJSW availability suggested minimal differences, the possibility of this bias cannot be excluded. In the context of multi-joint OA worsening, this selection toward a knee-predominant cohort may limit generalizability to general populations and raises the possibility that observed hip worsening reflects biomechanical compensation secondary to knee OA, rather than independent multi-joint disease progression. Finally, while the study aimed to identify associations with combined OA worsening, the exploratory nature of the subgroup comparisons and the relatively small size of the combined worsening group, and the inclusion of small subgroups with different underlying conditions, such as RA, warrant cautious interpretation. These findings should be considered as a first exploration and require validation in larger cohorts.

Despite the limitations, a major strength of this study is the use of a large, well-characterized cohort with longitudinal radiographic data on multiple joints spanning 48 months. This allowed for the identification and comparison of distinct OA worsening patterns across hip and knee joints, including the relatively understudied subgroup with combined OA worsening. To our knowledge, this is the first in-depth analysis to explore predictors of combined OA worsening using both univariate and multivariate approaches. The multinomial regression model provided new insights into associations across worsening groups, contributing to a more nuanced understanding of OA heterogeneity. This study also benefits from the inclusion of a broad set of baseline predictors, encompassing demographic, clinical, and joint structural variables. This comprehensive approach enabled the analysis or potential systemic and joint-specific factors associated with combined OA worsening.

For individuals with combined hip and knee OA worsening, our findings provide a foundation for more refined approaches to patient stratification and overall OA modeling. To build on this foundation, future work will require expanded datasets that capture more detailed, joint-level information. One important area for further investigation is the distinction between unilateral and bilateral involvement, both at baseline and when defining worsening groups. Although our analyses were conducted at the participant level, Supplementary Table S2–S4 highlight left-right differences that point to potential joint-level interplay. As the field moves forward, expanding datasets to incorporate joint-level detail, more refined structural and systemic variables such as comorbidities, and additional OA joint sites such as the hands will be essential for constructing a clearer and more comprehensive picture of multi-joint OA worsening. Such efforts will help determine whether patterns we observed represent distinct OA pathways or reflect the cumulative influence of single-joint processes. Additionally, collecting datasets with extensive information on pain trajectories is essential to examine if these differences are also apparent in individuals with symptomatic OA [48].

In conclusion, our findings suggest that individuals with multi-joint hip and knee OA worsening are different from individuals with no or isolated joint worsening, though these differences might reflect the accumulation of known single-joint OA risk factors rather than a separate clinical trajectory. While the evidence for a distinct subgroup remains unclear, recognizing individuals at risk of OA worsening in multiple joints and prioritizing targeted data collection in this population is important for both clinical care and research. Individuals with OA affecting multiple joints often experience greater functional impairment, reduced mobility, and higher healthcare utilization. Recognizing and characterizing these subgroups is critical not only for improving individual patient outcomes but also for informing public health strategies aimed at reducing the overall burden of OA.

## Author contributions

CRediT Classification:

Myrthe A. van den Berg: Conceptualization, data curation, formal analysis, funding acquisition, investigation, methodology, project administration, software, validation, visualization, writing - original draft preparation.

Egor Panfilov: Conceptualization, data curation, methodology, writing-review & editing.

Sita M.A. Bierma-Zeinstra: conceptualization, writing-review & editing.

Jesse H. Krijthe: conceptualization, supervision, writing-review & editing.

Rintje Agricola: conceptualization, funding acquisition, supervision, writing-review & editing.

Aleksei Tiulpin: Conceptualization, data curation, funding acquisition, methodology, supervision, writing-review & editing.

All authors have read and approved the final submitted manuscript.

## Role of the funding source

The authors are financially supported by the 10.13039/100018286Dutch Arthritis Society (grant no. 18-2-203 and 21-1-205), the 10.13039/501100003246Dutch Research Council (10.13039/501100003246NWO Veni grant scheme no. 09150161910071), the Erasmus MC, University Medical Center, Rotterdam (Erasmus MC Fellowship), and the 6GESS Profiling Research Programme (10.13039/501100002341Research Council of Finland, project 336449). This publication is part of the Flagship Healthy Joints which is financed by Convergence Health and Technology.

For the 10.13039/100019120OAI dataset, the cohort, clinical data and image acquisitions used in these analyses were fund as the Osteoarthritis Initiative through a public-private partnership comprised of five contracts (N01-AR-2-2258; N01-AR-2-2259; N01-AR-2 2260; N01-AR-2-2261; N01-AR-2-2262) funded by the 10.13039/100000002National Institutes of Health, a branch of the Department of Health and Human Services, and conducted by the 10.13039/100019120OAI Study Investigators. Private funding partners include 10.13039/100004334Merck Research Laboratories; 10.13039/100008272Novartis Pharmaceuticals Corporation, GlaxoSmithKline; and 10.13039/100004319Pfizer, Inc. Private sector funding for the 10.13039/100019120OAI is managed by the 10.13039/100000009Foundation for the National Institutes of Health.

## Competing interests

The authors declare they have no competing interests.
