# Supplementary material for: Is multi-joint hip and knee osteoarthritis more than the sum of its parts?
Source: Osteoarthr Cartil Open. 2026 May 14;8(2):100814. doi: 10.1016/j.ocarto.2026.100814 (PMC13214521; doi:10.1016/j.ocarto.2026.100814)
Supplement: Multimedia component 1 [file mmc1.docx]

**Supplementary material**

**Table S1:** Demographics and baseline characteristics for excluded and included
participants after exclusion for the univariate analysis. For included participants, the percentage of missing data is also shown.

| **Variable** | **Full set** | **Excluded** | **Included** | **% Missing data** |
| --- | --- | --- | --- | --- |
| Number of participants, N | 4.796 | 2,838 | 1,958 |  |
|  |  |  |  |  |
| **Demographics** |  |  |  |  |
| Age [years], mean (sd) | 61.16 (9.19) | 61.09 (9.34) | 61.26 (8.97) | 0.0% |
| BMI [kg/m^2^], mean (sd) | 28.62 (4.84) | 28.49 (5.00) | 28.81 (4.59) | 0.1% |
| Female participants, N (%)'' | 2.804 (58.5) | 1,670 (58.8) | 1,134 (57.9) | 0.0% |
|  |  |  |  |  |
| **Clinical factors** |  |  |  |  |
| Hip pain present, N (%)'' | 2.651 (55.3) | 1,600 (56.4) | 1,051 (53.7) | 0.2% |
| Knee pain present, N (%)'' | 4.091 (85.3) | 2,426 (85.5) | 1,655 (84.5) | 0.0% |
| Knee injury status, N (%)'' |  |  |  | 0.5% |
| No injury | 2.433 (50.7) | 1,475 (52.0) | 958 (48.9) |  |
| Injury without surgery | 1.224 (25.5) | 721 (25.4) | 503 (25.7) |  |
| Surgical intervention | 1.077 (22.5) | 601 (21.2) | 476 (24.3) |  |
| Asthma, N (%)'' | 396 (8.3) | 254 (8.9) | 151 (7.7) | 2.6% |
| Lung disease, N (%)'' | 105 (2.2) | 73 (2.6) | 32 (1.6) | 2.3% |
| Diabetes, N (%)'' | 362 (7.5) | 216 (7.6) | 146 (7.5) | 1.8% |
| RA, N (%)'' | 66 (1.4) | 35 (1.2) | 31 (1.6) | 16.0% |
| Abdominal circumference [cm], mean (sd) | 102.43 (12.87) | 101.85 (13.25) | 103.30 (12.24) | 3.8% |
|  |  |  |  |  |
| **OA history and genetic indicators** |  |  |  |  |
| Hand OA, N (%)'' | 783 (16.3) | 482 (17.0) | 301 (15.4) | 3.7% |
| Family member with hip replacement, N (%)'' | 429 (8.9) | 253 (8.9) | 176 (9.0) | 0.9% |
| Family member with knee replacement, N (%)'' | 663 (13.8) | 391 (13.8) | 272 (13.9) | 1.6% |
|  |  |  |  |  |
| **Structural joint features** |  |  |  |  |
| Hip OA status in worst hip, N (%)'' |  |  |  | 0.0% |
| No OA (grade 0) | 3314 (69.1) | 1,829 (64.4) | 1,485 (75.8) |  |
| Possible OA (grade 1) | 605 (12.6) | 309 (10.9) | 296 (15.1) |  |
| Definite OA (grade 2) | 398 (8.3) | 221 (7.8) | 177 (9.0) |  |
| Knee OA status in worst knee, N (%)'' |  |  |  | 0.0% |
| KL grade 0 | 1243 (25.9) | 977 (34.4) | 266 (13.6) |  |
| KL grade 1 | 686 (14.3) | 441 (15.5) | 245 (13.6) |  |
| KL grade 2 | 1336 (27.9) | 580 (20.4) | 756 (38.6) |  |
| KL grade 3 | 874 (18.2) | 344 (12.1) | 530 (27.1) |  |
| KL grade 4 | 292 (6.1) | 131 (4.6) | 161 (8.2) |  |
| Left hip |  |  |  |  |
| AA [degrees], mean (sd) | 49.11 (12.29) | 48.78 (12.15) | 49.59 (12.48) | 0.0% |
| LCEA [degrees], mean (sd) | 35.99 (5.82) | 36.18 (5.87) | 35.71 (5.73) | 0.1% |
| Right hip |  |  |  |  |
| AA [degrees], mean (sd) | 47.71 (11.49) | 47.49 (11.47) | 48.02 (11.51) | 0.0% |
| LCEA [degrees], mean (sd) | 34.99 (5.71) | 35.18 (5.79) | 34.71 (5.58) | 0.0% |
| Left knee |  |  |  |  |
| Flexion contracture/hyperextension  (contracture positive) [degrees], mean (sd) | -0.24 (4.07) | -0.42 (3.98) | 0.02 (4.19) | 4.1% |
| Alignment angle  (Valgus negative) [degrees], mean (sd) | -0.61 (3.7) | -0.64 (3.58) | -0.56 (3.87) | 4.1% |
| Right knee |  |  |  |  |
| Flexion contracture/hyperextension  (contracture positive) [degrees], mean (sd) | 0.32 (4.2) | 0.12 (4.23) | 0.61 (4.15) | 4.0% |
| Alignment angle  (Valgus negative) [degrees], mean (sd) | -0.57 (3.69) | -0.65 (3.60) | -0.46 (3.82) | 4.0% |

*BMI: Body Mass Index, AA: alpha angle, LCEA: lateral center edge angle, RHOA: radiographic hip osteoarthritis grade, RKOA: radiographic knee osteoarthritis grade, JSW: joint space width, OA: osteoarthritis, RA: rheumatoid arthritis.*
*Percentages are relative to the number of excluded/included participants. The column indicating missing data is to the included dataset.*

**Table S2:** Descriptive statistics and found odds-ratios with 95%-CI for logistic regression model predicting exclusion based on mJSW availability.

| **Variable** | **With mJSW measurements** | **Without mJSW measurements** | **SMD** | **aOR (95% CI)** |
| --- | --- | --- | --- | --- |
| Number of participants N | 1.944 | 2.040 |  |  |
| Age [years], mean (sd) | 61.25 (8.95) | 60.54 (9.28) | 0.079 | 1.01 (1.00 - 1.02) |
| BMI [kg/m^2^], mean (sd) | 28.82 (4.58) | 27.60 (4.54) | 0.266 | 0.98 (0.96 - 0.99) |
| Female participants, N (%) | 1.123 (57.8) | 1.163 (57.0) | 0.015 | 0.86 (0.75 - 0.99) |
| Hip pain present, N (%) | 1.049 (54.0) | 1.139 (55.8) | 0.038 | 1.05 (0.92 - 1.21) |
| Knee pain present, N (%) | 1.645 (84.6) | 1.734 (85.0) | 0.011 | 1.60 (1.32 - 1.94) |
| Hip OA status in worst hip, N (%) |  |  | 0.061 | 1.04 (0.93 - 1.16) |
| No OA (grade 0) | 1.474 (75.8) | 1.585 (77.7) |  |  |
| Possible OA (grade 1) | 295 (15.2) | 266 (13.0) |  |  |
| Definite OA (grade 2) | 175 ( 9.0) | 189 ( 9.3) |  |  |
| Knee OA status in worst knee, N (%) |  |  | 0.843 | 0.52 (0.49 - 0.55) |
| KL grade 0 | 264 (13.6) | 899 (44.1) |  |  |
| KL grade 1 | 244 (12.6) | 383 (18.8) |  |  |
| KL grade 2 | 749 (38.5) | 449 (22.0) |  |  |
| KL grade 3 | 527 (27.1) | 216 (10.6) |  |  |
| KL grade 4 | 160 ( 8.2) | 93 ( 4.6) |  |  |

*aOR: adjusted odds ratio, CI: confidence interval, BMI: Body Mass Index, RHOA: radiographic hip osteoarthritis grade, RKOA: radiographic knee osteoarthritis grade, mJSW: minimum joint space width, OA: osteoarthritis,*
*Percentages are relative to the number of participants in the respective categories. The column indicating missing data is to the included dataset.*

**Table S3:** Descriptive statistics on considered predictors for excluded and included participants for the multivariate analysis.

| **Variable** | **Excluded** | **% Missing data** | **Included** | **SMD** |
| --- | --- | --- | --- | --- |
| Number of participants, N (%)' | 151 |  | 1.807 |  |
| Worsening group, N (%)” |  | 0% |  | 0.24 |
| No | 64 (42.4) |  | 766 (42.4) |  |
| Hip-only | 37 (24.5) |  | 295 (16.3) |  |
| Knee-only | 32 (21.2) |  | 519 (28.7) |  |
| Combined | 18 (11.9) |  | 227 (12.6) |  |
| **Demographics** |  |  |  |  |
| Age [years], mean (sd) | 57.51 (9.25) | 0% | 61.57 (8.87) | 0.45 |
| BMI [kg/m^2^], mean (sd) | 26.87 (5.06) | 1% | 28.97 (4.51) | 0.44 |
| Female participants, N (%)'' | 93 (61.6) | 0% | 1041 (57.6) | 0.08 |
|  |  |  |  |  |
| **Clinical factors** |  |  |  |  |
| Hip pain present, N (%)'' | 54 (35.8) | 0% | 997 (55.2) | 0.50 |
| Knee pain present, N (%)'' | 68 (45.0) | 0% | 1587 (87.8) | 1.02 |
| Knee injury status, N (%)'' | 36 (23.8) | 14% | 943 (52.2) | 0.78 |
|  |  |  |  |  |
| **OA history and genetic indicators** |  |  |  |  |
| Family member with hip replacement, N (%)'' | 12 ( 7.9) | 12% | 164 ( 9.1) | 0.52 |
| Family member with knee replacement, N (%)'' | 4 ( 2.6) | 21% | 268 (14.8) | 0.84 |
|  |  |  |  |  |
| **Structural joint features** |  |  |  |  |
| Hip OA status in worst hip, N (%)'' |  | 0% |  | 0.10 |
| No OA (grade 0) | 120 (79.5) |  | 1365 (75.5) |  |
| Possible OA (grade 1) | 20 (13.2) |  | 276 (15.3) |  |
| Definite OA (grade 2) | 11 ( 7.3) |  | 166 ( 9.2) |  |
| Knee OA status in worst knee, N (%)'' |  | 0% |  | 0.82 |
| KL grade 0 | 63 (41.7) |  | 203 (11.2) |  |
| KL grade 1 | 25 (16.6) |  | 220 (12.2) |  |
| KL grade 2 | 35 (23.2) |  | 721 (39.9) |  |
| KL grade 3 | 21 (13.9) |  | 509 (28.2) |  |
| KL grade 4 | 7 ( 4.6) |  | 154 ( 8.5) |  |
| Left knee |  |  |  |  |
| Alignment angle  (Valgus negative) [degrees], mean (sd) | -0.42 (3.87) | 53% | -0.57 (3.87) | 0.02 |
| mJSW [mm], mean (sd) | 4.47 (1.14) | 0% | 4.15 (1.35) | 0.25 |
| Right knee |  |  |  |  |
| Alignment angle  (Valgus negative) [degrees], mean (sd) | -0.51 (3.63) | 53% | -0.45 (3.83) | 0.04 |
| mJSW [mm], mean (sd) | 4.49 (1.18) | 0% | 4.23 (1.31) | 0.21 |
| Left hip |  |  |  |  |
| mJSW [mm], mean (sd) | 3.59 (0.60) | 0% | 3.50 (0.59) | 0.14 |
| Right hip |  |  |  |  |
| mJSW [mm], mean (sd) | 3.64 (0.58) | 0% | 3.56 (0.60) | 0.13 |

*BMI: Body Mass Index, RHOA: radiographic hip osteoarthritis grade, RKOA: radiographic knee osteoarthritis grade, OA: osteoarthritis, SMD: standardized mean difference between Included and Excluded. Percentages are relative to the number of excluded/included participants. The column indicating missing data relates to the excluded dataset.*


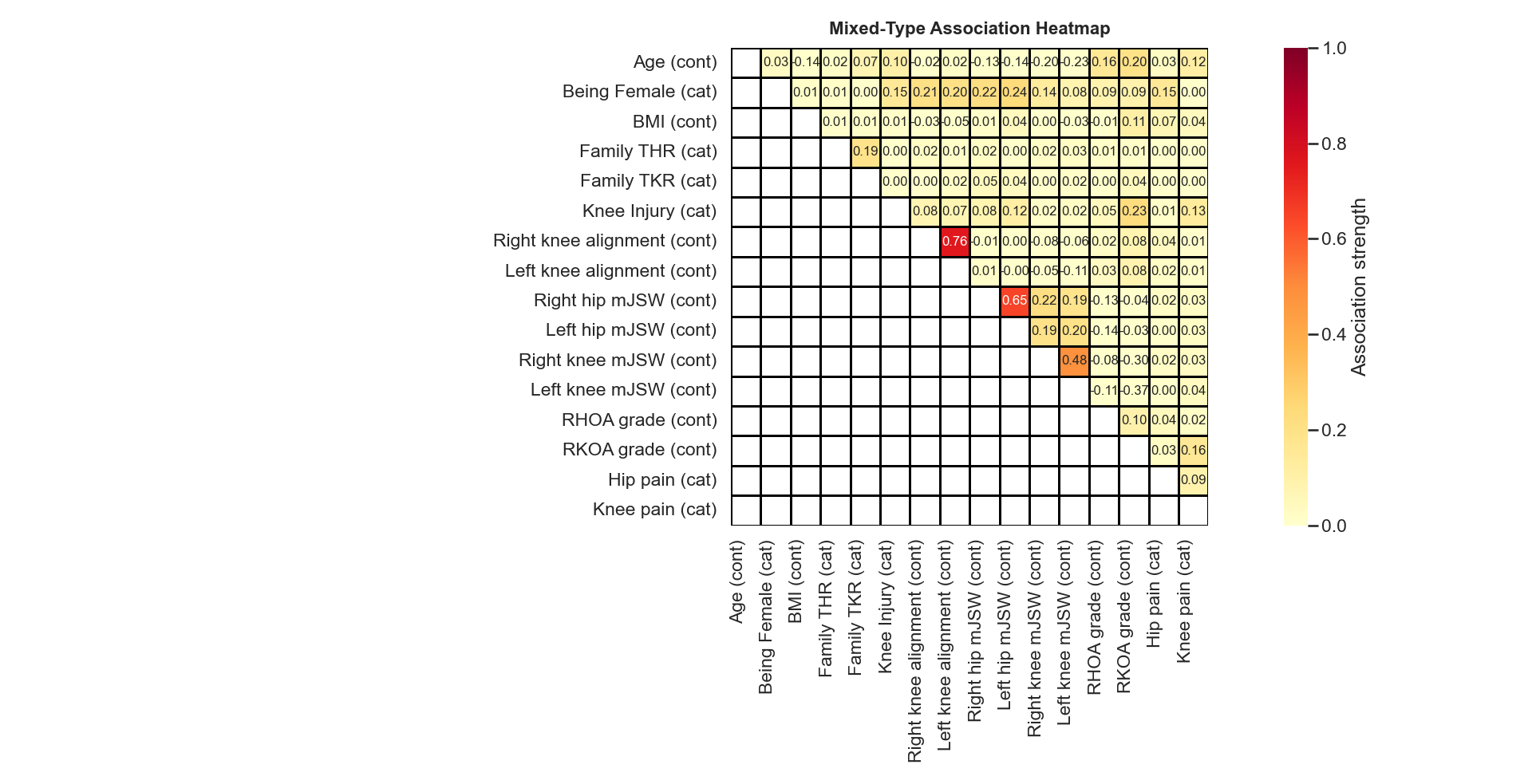


**Figure S1:** Mixed-type association heatmap between the predictors used in the multinomial regression models. Continuous-continuous associations are represented by Spearman correlation coefficients, categorical-continuous associations by the correlation ratio (η), and categorical-categorical associations by Cramér’s V.

*cat: categorical variable; cont: continuous variable.*

**Table S4:** Descriptions of baseline OA status within the included study population per worsening subgroup.

| **Variable** | **Worsening group** | | | |
| --- | --- | --- | --- | --- |
|  | **No** | **Hip-only** | **Knee-only** | **Combined** |
|  | **N (%)** | **N (%)** | **N (%)** | **N (%)** |
| Number of participants | 830 | 332 | 551 | 245 |
| ROA ≥ 2 in |  |  |  |  |
| 0 joints | 253 (30.5) | 93 (28.0) | 85 (15.4) | 45 (18.4) |
| 1 joint | 227 (27.3) | 99 (29.8) | 155 (28.1) | 61 (24.9) |
| 2 joints | 317 (38.2) | 119 (35.8) | 281 (51.0) | 124 (50.6) |
| 3 joints | 20 (2.4) | 16 (4.8) | 23 (4.2) | 11 (4.5) |
| 4 joints | 13 (1.6) | 5 (1.5) | 7 (1.3) | 4 (1.6) |
| ROA > 0 in |  |  |  |  |
| at least one hip and one knee | 166 (20.0) | 79 (23.8) | 125 (22.7) | 57 (23.3) |
| right hip & left hip | 113 (13.6) | 41 (12.3) | 83 (15.1) | 30 (12.2) |
| right knee & left knee | 507 (61.1) | 203 (61.1) | 413 (75.0) | 179 (73.1) |
| right hip & right knee | 117 (14.1) | 58 (17.5) | 90 (16.3) | 36 (14.7) |
| left hip & left knee | 122 (14.7) | 46 (13.9) | 94 (17.1) | 43 (17.6) |
| right hip & left knee | 124 (14.9) | 54 (16.3) | 91 (16.5) | 35 (14.3) |
| left hip & right knee | 119 (14.3) | 49 (14.8) | 93 (16.9) | 47 (19.2) |

*ROA: radiographic OA grade (0-2 for the hip, 0-4 (KLG) for the knee)*. *Percentages are relative to the total worsening group size*

**Table S5:** Median joint space width narrowing in 48 months per joint per subgroup based on ROA grade at baseline in a particular joint.

|  |  |  | | | **Right hip** | | | **Left hip** | | | **Right knee** | | | **Left knee** | |
| --- | --- | --- | --- | --- | --- | --- | --- | --- | --- | --- | --- | --- | --- | --- | --- |
|  |  | **N** | | **Median JSN  [mm]** | | **IQR  [mm]** | **Median JSN  [mm]** | | **IQR  [mm]** | **Median JSN  [mm]** | | **IQR  [mm]** | **Median JSN  [mm]** | | **IQR  [mm]** |
| **Overall** |  | 1958 | | -0.083 | | 0.373 | -0.085 | | 0.366 | -0.259 | | 0.706 | -0.29 | | 0.766 |
|  |  |  | |  | |  |  | |  |  | |  |  | |  |
| **Baseline ROA  grade in** | |  | |  | |  |  | |  |  | |  |  | |  |
| Right hip | 0 | | 1592 | -0.086 | | 0.373 | -0.090 | | 0.375 | -0.259 | | 0.706 | -0.284 | | 0.752 |
|  | 1 | | 254 | -0.053 | | 0.335 | -0.060 | | 0.352 | -0.286 | | 0.713 | -0.326 | | 0.805 |
|  | 2 | | 112 | -0.104 | | 0.474 | -0.036 | | 0.317 | -0.187 | | 0.598 | -0.287 | | 0.730 |
|  |  | |  |  | |  |  | |  |  | |  |  | |  |
| Left hip | 0 | | 1584 | -0.084 | | 0.374 | -0.086 | | 0.370 | -0.251 | | 0.693 | -0.281 | | 0.749 |
|  | 1 | | 261 | -0.076 | | 0.351 | -0.063 | | 0.287 | -0.289 | | 0.747 | -0.317 | | 0.798 |
|  | 2 | | 113 | -0.120 | | 0.454 | -0.154 | | 0.454 | -0.306 | | 0.734 | -0.306 | | 0.836 |
|  |  | |  |  | |  |  | |  |  | |  |  | |  |
| Right knee | 0 | | 448 | -0.078 | | 0.336 | -0.089 | | 0.354 | -0.236 | | 0.575 | -0.223 | | 0.589 |
|  | 1 | | 338 | -0.080 | | 0.381 | -0.093 | | 0.368 | -0.176 | | 0.562 | -0.236 | | 0.700 |
|  | 2 | | 744 | -0.084 | | 0.379 | -0.075 | | 0.352 | -0.285 | | 0.738 | -0.268 | | 0.780 |
|  | 3 | | 351 | -0.091 | | 0.388 | -0.107 | | 0.397 | -0.415 | | 0.857 | -0.410 | | 0.855 |
|  | 4 | | 77 | -0.094 | | 0.311 | -0.047 | | 0.353 | 0.000 | | 0.247 | -0.548 | | 1.076 |
|  |  | |  |  | |  |  | |  |  | |  |  | |  |
| Left knee | 0 | | 474 | -0.109 | | 0.386 | -0.062 | | 0.375 | -0.224 | | 0.546 | -0.219 | | 0.544 |
|  | 1 | | 331 | -0.090 | | 0.383 | -0.133 | | 0.391 | -0.229 | | 0.668 | -0.289 | | 0.681 |
|  | 2 | | 691 | -0.064 | | 0.374 | -0.081 | | 0.327 | -0.235 | | 0.668 | -0.259 | | 0.779 |
|  | 3 | | 377 | -0.050 | | 0.331 | -0.063 | | 0.385 | -0.397 | | 0.927 | -0.566 | | 1.013 |
|  | 4 | | 85 | -0.142 | | 0.404 | -0.096 | | 0.403 | -0.477 | | 0.883 | -0.006 | | 0.503 |

*IQR: interquartile range, JSN: joint space width narrowing, ROA: radiographic OA grade (0-2 for the hip, 0-4 (KLG) for the knee)*

**Table S6:** Number of progressing joints in 48 months per joint per subgroup based on ROA grade at baseline.

|  |  |  | **OA worsening in** | | | | | | | |
| --- | --- | --- | --- | --- | --- | --- | --- | --- | --- | --- |
|  |  |  | **Right hip** | | **Left hip** | | **Right knee** | | **Left knee** | |
|  |  | **N** | **N** | **%** | **N** | **%** | **N** | **%** | **N** | **%** |
| **Overall** |  | 1,958 | 294 | 15% | 309 | 16% | 496 | 25% | 531 | 27% |
|  |  |  |  |  |  |  |  |  |  |  |
| **Baseline ROA grade in** |  |  |  |  |  |  |  |  |  |  |
| Right hip | 0 | 1,592 | 272 | 17% | 254 | 16% | 403 | 25% | 427 | 27% |
|  | 1 | 254 | 40 | 16% | 41 | 16% | 68 | 27% | 73 | 29% |
|  | 2 | 112 | 27 | 24% | 14 | 13% | 25 | 22% | 31 | 28% |
| Left hip | 0 | 1,584 | 273 | 17% | 247 | 16% | 390 | 25% | 424 | 27% |
|  | 1 | 261 | 42 | 16% | 36 | 14% | 74 | 28% | 77 | 30% |
|  | 2 | 113 | 24 | 21% | 26 | 23% | 32 | 28% | 30 | 27% |
| Right knee | 0 | 448 | 70 | 16% | 74 | 17% | 90 | 20% | 88 | 20% |
|  | 1 | 338 | 57 | 17% | 54 | 16% | 69 | 20% | 85 | 25% |
|  | 2 | 744 | 138 | 19% | 105 | 14% | 204 | 27% | 212 | 28% |
|  | 3 | 351 | 66 | 19% | 61 | 17% | 121 | 34% | 117 | 33% |
|  | 4 | 77 | 8 | 10% | 15 | 19% | 12 | 16% | 29 | 38% |
| Left knee | 0 | 474 | 82 | 17% | 84 | 18% | 91 | 19% | 82 | 17% |
|  | 1 | 331 | 56 | 17% | 56 | 17% | 79 | 24% | 80 | 24% |
|  | 2 | 691 | 126 | 18% | 93 | 13% | 165 | 24% | 187 | 27% |
|  | 3 | 377 | 58 | 15% | 60 | 16% | 128 | 34% | 165 | 44% |
|  | 4 | 85 | 17 | 20% | 16 | 19% | 33 | 39% | 17 | 20% |

*OA: osteoarthritis, ROA: radiographic OA grade (0-2 for the hip, 0-4 (KLG) for the knee)*

**Table S7:** Estimated adjusted odds ratios and 95% confidence intervals for the multinomial regression models for both the complete case analysis and the imputation analysis.

|  | **Comparison 1  (No vs Combined)** | | | | | **Comparison 2  (Hip vs Combined)** | | | | | **Comparison 3  (No vs Combined)** | |
| --- | --- | --- | --- | --- | --- | --- | --- | --- | --- | --- | --- | --- |
|  | **Complete cases** | | **Imputation** | **Complete cases** | | | **Imputation** | | **Complete cases** | | | **Imputation** |
|  | **aOR  (95% CI)** | **aOR  (95% CI)** | | | **aOR  (95% CI)** | | | **aOR  (95% CI)** | | **aOR  (95% CI)** | | **aOR  (95% CI)** |
| **Baseline demographics** |  |  | | |  | | |  | |  | |  |
| **Age [SD]** | 1.10  (0.93-1.30) | 1.11  (0.94-1.31) | | | 1.07  (0.89-1.30) | | | 1.08  (0.89-1.30) | | 1.04  (0.88-1.24) | | 1.05  (0.89-1.25) |
| **BMI [SD]** | 1.11  (0.95-1.30) | 1.13  (0.96-1.32) | | | 1.21  (1.01-1.45) | | | 1.22  (1.02-1.46) | | 0.99  (0.84-1.17) | | 1.01  (0.86-1.20) |
| **Female sex (ref: Male)** | 1.41  (0.99-1.99) | 1.42  (1.01-1.99) | | | 0.96  (0.64-1.43) | | | 0.93  (0.63-1.37) | | 1.92  (1.34-2.76) | | 1.98  (1.38-2.82) |
| **Clinical features** |  |  | | |  | | |  | |  | |  |
| **Knee inury (ref: No)** | 1.19  (0.86-1.66) | 1.17  (0.84-1.61) | | | 1.12  (0.77-1.63) | | | 1.11  (0.77-1.60) | | 1.22  (0.87-1.73) | | 1.18  (0.84-1.66) |
| **Hip pain (ref: No)** | 0.95  (0.69-1.30) | 0.97 (0.71-1.32) | | | 0.90  (0.62-1.29) | | | 0.91  (0.64-1.30) | | 0.92  (0.66-1.29) | | 0.95  (0.68-1.31) |
| **Knee pain (ref: No)** | 1.40  (0.82-2.38) | 1.38  (0.82-2.32) | | | 1.36  (0.75-2.45) | | | 1.28  (0.72-2.26) | | 1.18  (0.67-2.07) | | 1.19  (0.69-2.06) |
| **Genetic indicators** |  |  | | |  | | |  | |  | |  |
| **Family member with  THR (ref: No)** | 1.22  (0.73-2.04) | 1.25  (0.75-2.06) | | | 1.17  (0.65-2.13) | | | 1.13  (0.64-2.01) | | 1.38  (0.80-2.39) | | 1.46  (0.85-2.51) |
| **Family member with  TKR (ref: No)** | 0.99  (0.64-1.53) | 0.97  (0.63-1.50) | | | 1.47  (0.87-2.49) | | | 1.51  (0.89-2.56) | | 0.80  (0.51-1.26) | | 0.77  (0.49-1.21) |
| **Structural joint features** |  |  | | |  | | |  | |  | |  |
| **Knee Alignment Angle  (valgus negative) [SD]** |  |  | | |  | | |  | |  | |  |
| **Right knee** | 1.13  (0.89-1.44) | 1.10  (0.88-1.39) | | | 1.25  (0.95-1.64) | | | 1.22  (0.94-1.59) | | 1.09  (0.85-1.38) | | 1.07  (0.84-1.35) |
| **Left knee** | 1.03  (0.81-1.31) | 1.04  (0.82-1.31) | | | 1.08  (0.82-1.43) | | | 1.08  (0.82-1.41) | | 0.92  (0.72-1.17) | | 0.91  (0.72-1.16) |
| **ROA status [1 grade]** |  |  | | |  | | |  | |  | |  |
| **Hip** | 1.27  (0.99-1.63) | 1.28 (1.00-1.63) | | | 0.87  (0.66-1.15) | | | 0.87  (0.67-1.14) | | 1.22  (0.94-1.58) | | 1.24  (0.96-1.60) |
| **Knee** | 1.38  (1.16-1.64) | 1.38  (1.17-1.64) | | | 1.36  (1.12-1.66) | | | 1.34  (1.11-1.62) | | 0.94  (0.79-1.14) | | 0.94  (0.79-1.13) |
| **mJSW [SD]** |  |  | | |  | | |  | |  | |  |
| **Right hip** | 1.50  (1.22-1.84) | 1.53 (1.25-1.87) | | | 0.97  (0.77-1.22) | | | 1.00  (0.80-1.24) | | 1.54  (1.25-1.91) | | 1.59  (1.29-1.96) |
| **Left hip** | 1.56  (1.26-1.92) | 1.55  (1.26-1.90) | | | 1.11  (0.87-1.41) | | | 1.12  (0.89-1.40) | | 1.48  (1.19-1.84) | | 1.49  (1.20-1.84) |
| **Right knee** | 0.99  (0.84-1.17) | 1.01  (0.86-1.19) | | | 1.02  (0.84-1.24) | | | 1.03  (0.85-1.24) | | 0.81  (0.68-0.96) | | 0.82  (0.70-0.97) |
| **Left knee** | 1.06  (0.90-1.26) | 1.04  (0.88-1.23) | | | 1.08  (0.88-1.32) | | | 1.03  (0.85-1.25) | | 0.99  (0.84-1.18) | | 0.98  (0.83-1.16) |

Imputation was performed using multiple imputation by chained equations (MICE), generating 20 imputed datasets with 10 iterations each.

*aOR: adjusted odds ratio, BMI: Body Mass Index, CI: confidence interval, ROA: radiographic osteoarthritis, mJSW: minimum joint space width, THR: total hip replacement, TKR: total knee replacement*
